# Fast Gene Ontology based clustering for microarray experiments

**DOI:** 10.1186/1756-0381-1-11

**Published:** 2008-11-21

**Authors:** Kristian Ovaska, Marko Laakso, Sampsa Hautaniemi

**Affiliations:** 1 Computational Systems Biology Laboratory, Institute of Biomedicine and Genome-Scale Biology Program, Biomedicum Helsinki, University of Helsinki, PO Box 63 (Haartmaninkatu 8), 00014 UNIVERSITY OF HELSINKI, Finland

## Abstract

**Background:**

Analysis of a microarray experiment often results in a list of hundreds of disease-associated genes. In order to suggest common biological processes and functions for these genes, Gene Ontology annotations with statistical   testing are widely used. However, these analyses can produce a very large number of significantly altered biological processes. Thus, it is often challenging to interpret GO results and identify novel testable biological hypotheses.

**Results:**

We present fast software for advanced gene annotation using semantic similarity for Gene Ontology terms combined with clustering and heat map visualisation. The methodology allows rapid identification of genes   sharing the same Gene Ontology cluster.

**Conclusion:**

Our R based semantic similarity open-source package has a speed advantage of over 2000-fold compared to existing implementations. From the resulting hierarchical clustering dendrogram genes sharing a GO term can be identified, and their differences in the gene expression patterns can be seen from the heat map. These methods facilitate advanced annotation of genes resulting from data analysis.

## Background

A microarray experiment may result in hundreds of differentially expressed genes that are subject to interpretation and further analysis. As analysing these lists gene-by-gene is tedious and error prone, the genes in the lists are routinely annotated using Gene Ontology (GO) with an aim to identify statistically significant biological processes or pathways [1]. However, statistical analysis of GO annotations can produce a very large number of significantly enriched or down-regulated biological processes. Thus, it is often challenging to interpret GO results and identify novel testable biological hypotheses.

The GO project provides a species-independent controlled vocabulary for describing gene products (an RNA or protein product encoded by a gene) in terms of their biological processes, cellular components and molecular functions [1]. The GO annotations are carried out by curators of several bioinformatics databases, so the GO database is constantly updated. The ontology defines terms that are linked together to form a directed acyclic graph. Gene products are annotated with a number of ontology terms. Annotation with a given term also implies annotation with all ancestors of the term.

In this study we present methodology and software to cluster genes based on their biological functionality using GO annotations. Integral part of the methodology is the ability to rapidly compute pair-wise distances between the gene annotation similarities.

Two approaches to gene similarity computation are graph structure -based (GS) and information content -based (IC) measures. GS-based methods use the hierarchical structure of GO in computing gene similarity. IC-based methods additionally consider the *a priori *probabilities, or information contents, of GO terms in a reference gene set. IC-based measures have been found to perform better than pure graph-based measures [2,3].

Czekanowski-Dice similarity [4] is a GS-based method. Distance of genes *G*_1 _and *G*_2 _is defined as

$$d(G_{1},G_{2})=\frac{\#(GO(G_{1})\Delta GO(G_{2}))}{\#(GO(G_{1})\cup GO(G_{2}))+\#(GO(G_{1})\cap GO(G_{2}))},$$

where Δ is the symmetric set difference, # is the number of elements in a set and *GO*(*G*_*i*_) is the set of *GO *annotations for gene *G*_*i*_. Similarity can be defined as 1 - *d*(*G*_1_, *G*_2_).

In Kappa statistics [5], each gene is represented as a binary vector (*g*_1_,...,*g*_*N*_), where *g*_*i *_is 1 if the gene is annotated with the GO term *g*_*i *_and 0 otherwise. *N *is the total number of GO terms under consideration.

Similarity of genes *G*_1 _and *G*_2 _is defined as

$$K_{G_{1},G_{2}}=\frac{O_{G_{1},G_{2}}-A_{G_{1},G_{2}}}{1-A_{G_{1},G_{2}}},$$

where $O_{G_{1},G_{2}}$ represents observed co-occurrence of GO terms and $A_{G_{1},G_{2}}$ represents random co-occurrence. $O_{G_{1},G_{2}}$ is the relative frequency of agreeing locations in the two binary vectors, i.e., locations that are either both 0 or both 1. $A_{G_{1},G_{2}}$ is the expected relative frequency of such locations if the binary vectors were random, taking into account the observed probabilities of 0's and 1's.

The following discussion considers IC-based similarity measures. The information content of a GO term is computed by the frequency of the term occurring in annotations; a rarely used term contains a greater amount of information. Probability for observing a term *t *is defined as $p(t)=\frac{\text{Freq}(\text{t})}{\text{MaxFreq}}$, where MaxFreq is the maximum frequency of all terms [6]. The information content for a term *t *is given as *IC*(*t*) = -log_2_*p*(*t*). Probabilities can be estimated from a corpus of annotations, such as the Gene Ontology database.

Several related similarity metrics are based on the most informative common ancestor (MICA) of two GO terms and were introduced in the context of GO by Lord et al. [7]. To compute the semantic similarity between terms *t*_1 _and *t*_2_, we first find the most informative common ancestor *A *of *t*_1 _and *t*_2_, i.e., *A *is a term that is an ancestor of both *t*_1 _and *t*_2 _and has the maximum *IC *among common ancestors *CommonAnc*(*t*_1,_*t*_2_) of the terms. Now, the Resnik similarity [8] is defined as

Sim_*Resnik*_(*t*_1_, *t*_2_) = *IC*(*A*).

Several other measures are defined that also take the information contents of *t*_1 _and *t*_2 _into account. The Lin measure [9] is defined as

(1) $${\text{Sim}}_{Lin}(t_{1},t_{2})=\frac{2IC(A)}{IC(t_{1})+IC(t_{2})}.$$

Jiang and Conrath [10] define a semantic distance metric as

*d*_*JC*_(*t*_1_, *t*_2_) = *IC*(*t*_1_) + *IC*(*t*_2_) - 2*IC*(*A*).

The corresponding similarity measure for *d*_*JC*_(*t*_1_, *t*_2_) [6] is given by

$${\text{Sim}}_{JC}(t_{1},t_{2})=\frac{1}{d_{JC}(t_{1},t_{2})+1}.$$

Finally, the Relevance measure [11] that combines Lin's and Resnik's measures is defined as

$${\text{Sim}}_{Rel}(t_{1},t_{2})=\underset{t\in CommonAnc(t_{1},t_{2})}{\max}\frac{2\log p(t)(1-p(t))}{\log p(t_{1})+\log p(t_{2})}=\frac{2IC(A)(1-p(A))}{IC(t_{1})+IC(t_{2})}.$$

The MICA-based measures can be modified to take into account so called disjunctive ancestor terms [6]. Two ancestors *a*_1 _and *a*_2 _of a term *t *are disjunctive if there are independent paths from *a*_1 _to *t *and from *a*_2 _to *t*. Such ancestors represent distinct interpretations of the term *t*. In the GraSM enhancement, all common disjunctive ancestors of terms *t*_1 _and *t*_2 _are considered when computing Sim(*t*_1,_*t*_2_) [6]. GraSM modifies the computation of *IC*(*A*) and can be applied to the Resnik, Lin and Jiang-Conrath measures. 

After computing the pair-wise term similarities, the next step in MICA-based measures is to calculate the similarity between genes *G*_1 _and *G*_2_. This can be done in several ways and our package supports three most commonly used methods. In the two simplest methods, the maximum or the mean of pair-wise GO term similarities between annotation sets of *G*_1 _and *G*_2 _is used as the similarity value [12]. That is, when *G*_1 _is annotated with terms *t*_1_,...,*t*_*n *_and *G*_2 _with terms ${t^{\prime }}_{1},...,{t^{\prime }}_{m}$, pair-wise term similarities form an *n *× *m *matrix **S**. Now, Sim_*gene*_(*G*_1_, *G*_2_) is the maximum or the mean of the matrix. In the third method, similarity is defined as Sim_*gene*_(*G*_1_, *G*_2_) = max{*rowScore, columnScore*} [11], where

$$\begin{array}{ccc} rowScore=\frac{1}{n}\sum_{i=1}^{n}\underset{1\leq j\leq m}{\max}S_{ij} & \text{and} & columnScore=\frac{1}{m}\sum_{j=1}^{m}\underset{1\leq i\leq n}{\max}S_{ij}. \end{array}$$

In addition to MICA- and GraSM-based measures, we have implemented the cosine similarity and SimGIC measures. In cosine similarity [13], each gene *G *is represented as a vector (*w*_1_, *w*_2_,...,*w*_*N*_), where each *w*_*i *_is *IC*(*t*_*i*_) if *G *is annotated with the term *t*_*i*_, or 0 otherwise. *N *is the total number of GO terms under consideration. Similarity of genes *G*_1 _and *G*_2 _is defined as $\frac{G_{1}\cdot G_{2}}{\left|G_{1}||G_{2}\right|}$, where · is the dot product and |*v*| is the vector norm. This is the cosine of the angle between vectors *G*_1 _and *G*_2_. In the SimGIC (Graph Information Content) measure [3], similarity of genes *G*_1 _and *G*_2 _is defined as

$$\frac{\Sigma_{t\in GO(G_{1})\cap GO(G_{2})}IC(t)}{\Sigma_{t\in GO(G_{1})\cup GO(G_{2})}IC(t)},$$

where *GO*(*G*_*i*_) gives the GO annotations of gene *G*_*i*_. SimGIC is a hybrid of GS- and IC-based methods.

Given similarities between the genes we use hierarchical clustering with heat map presentation to visualise both semantic similarities and expression levels of the genes. First, similarity measures are converted to distances using *d*(*x*, *y*) = 1 - Sim(*x*, *y*) when the similarity range is [0, 1] (Czekanowski-Dice, Kappa, Lin, Jiang-Conrath, Relevance, Cosine, SimGIC) or using *d*(*x*, *y*) = 1/(Sim(*x*, *y*) + 1) when the range is [0, ∞) (Resnik). Second, a hierarchical clustering algorithm is run using the converted distances. The results are visualised as a dendrogram and heat map. The dendrogram is generated using the GO semantic distances and allows identification of clusters containing genes contributing to the same biological process. For each cluster we compute statistical significance with a permutation test. The heat map illustrates gene expression data obtained from microarray analysis. Thus, the visualisation framework integrates both functional gene expression levels to biological processes, which facilitates interpretation of the gene expression analysis results.

## Implementation

The semantic similarity package, csbl.go, is available for R [14]. The package computes similarities for arbitrary number of genes and supports the following measures: Czekanowski-Dice, Kappa, Resnik (with GraSM as an option), Jiang-Conrath (GraSM), Lin (GraSM), Relevance, Cosine and SimGIC. The MICA-based measures (Resnik, Lin, Jiang-Conrath, Relevance and GraSM enhancements) are implemented as a combination of R and C++ code; the four other measures are implemented in R. In addition to the regular R package, csbl.go is available as a component for Anduril [15], a framework for high-throughput data analysis we recently developed. The package is extensively tested and includes a user guide.

Similarity computation needs GO term probabilities for the reference gene set. We provide precomputed probability tables for *Homo sapiens*, *Saccharomyces cerevisiae*, *Caenorhabditis elegans*, *Drosophila melanogaster*, *Mus musculus *and *Rattus norvegicus*. The tables are computed based on all gene and protein annotations for the given organism found in the geneontology.org database. As GO is constantly updated and revised, we update the tables every six months. The package also has an option to use custom tables. The taxonomy ID of the organism is stored along with probability tables as metadata, which enables selection of a table by organism ID. The package also includes an option to compute GO term enrichment using Fisher's Exact Test [16].

## Results and discussion

We evaluated the package by using a performance benchmark and by applying the methods to microarray data from a testicular germ cell tumor study [17].

### Performance benchmark

We compared the performance of our package to two earlier introduced semantic similarity packages, SemSim 1.6.0 [18] and GOSim 1.1.5.1 [12]. The benchmark computes semantic similarities for GO term set sizes 50, 100 and 200. For csbl.go and SemSim, the measures Resnik, Jiang-Conrath, Lin and Relevance are used in the benchmark. GOSim does not support the Relevance measure so only the three other measures are used for it. The GraSM enhancement was not used in the benchmark as SemSim does not support GraSM.

The benchmark computes a symmetric *n *× *n *similarity matrix for the GO term sets. The three packages handle matrix computation in different ways. GOSim and csbl.go take a single term list and compute the symmetric matrix by computing half of of the pair-wise similarities (*n*^2^/2) and mirroring the matrix by the diagonal. SemSim takes two potentially different term lists and computes all *n*^2 ^pair-wise similarities. To compare the packages, we halved the execution times of SemSim in order to consider a situation where all packages perform *n*^2^/2 operations. The benchmark computes GO term similarities instead of gene similarities because the former is the most time-consuming part of similarity computation.

Benchmark results are in shown in Table 1. With the csbl.go package we obtained 2400- to 5000-fold (GOSim) and 2100- to 3000-fold (SemSim) speed gains. The speed gain achieved by csbl.go becomes more obvious with larger number of GO terms. For example, with 1000 terms SemSim and GOSim take more than 30 minutes while csbl.go takes less than one second.

**Table 1:** Benchmark results.

Measure	Number of GO terms	csbl.go	GOSim (vs. csbl.go)	SemSim (vs. csbl.go)
Resnik	50	0.002 s	4.9 s (2399 ×)	4.5 s (2158 ×)
Resnik	100	0.006 s	19.5 s (3219 ×)	17.4 s (2880 ×)
Resnik	200	0.024 s	77.8 s (3245 ×)	71.0 s (2963 ×)
Lin	50	0.002 s	7.4 s (3612 ×)	4.5 s (2167 ×)
Lin	100	0.007 s	29.5 s (4284 ×)	17.4 s (2528 ×)
Lin	200	0.024 s	117.8 s (4894 ×)	71.0 s (2950 ×)
Jiang-Conrath	50	0.002 s	7.4 s (3590 ×)	4.5 s (2157 ×)
Jiang-Conrath	100	0.007 s	29.5 s (4274 ×)	17.4 s (2525 ×)
Jiang-Conrath	200	0.023 s	117.5 s (5043 ×)	70.9 s (3043 ×)
Relevance	50	0.002 s	-	4.5 s (2062 ×)
Relevance	100	0.007 s	-	17.4 s (2400 ×)
Relevance	200	0.025 s	-	71.1 s (2866 ×)

Speed difference between csbl.go and other package is shown in parenthesis. Timings of csbl.go are shown with an accuracy of 1/1000 s.

### Case study

As a case study, we applied similarity measures to identify common GO classes for differentially expressed genes involved in testicular germ cell tumors (TGCTs). The TGCT microarray study here consists of five undifferentiated embryonal carcinoma samples and 12 differentiated testicular cell samples, which include both tumors and healthy samples [17].

We re-analysed the data set with the goal of finding differentially expressed genes (DEGs) between four undifferentiated samples (EC_0502, EC_0564, EC_1017 and EC_1740) and 10 differentiated samples (Cc_0915, N_9013, N_9014, N_0140, Ter_0691, Ter_0696, YST_0216, YST_0307, YST_0738, YST_2110). Three samples (EC_1838, Ter_1282 and Ter_2201) were excluded due to data quality problems. Data from the two-channel Agilent Human 1A were background corrected and processed with LOWESS [19]. DEGs were selected using t-test followed by false discovery rate correction [20]. We obtained 65 genes that have q-value below 0.1 and have also fold change of at least 1.5. We found GO annotations for 58 of the 65 genes using Ensembl version 50 [21]. Among the 58 genes, the median number of GO annotations per gene is eight.

**Figure 1:**
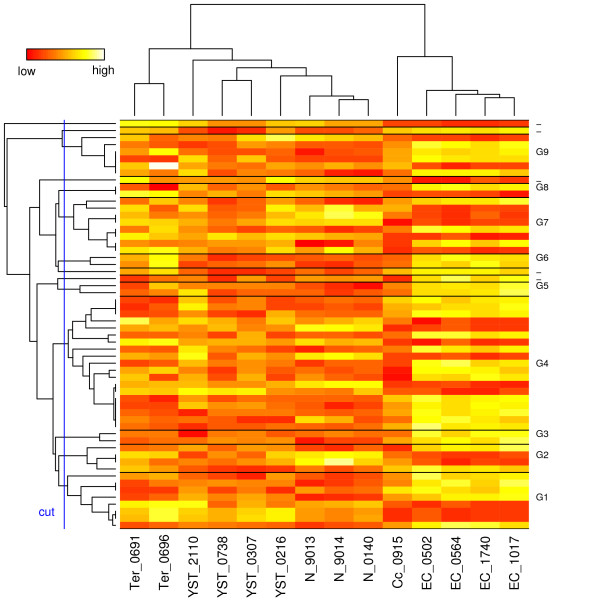
**GO heat map and clustering**. GO based clustering dendrogram of the selected genes (vertical axis) is visualised along with the expression patterns that are used to cluster the samples (horizontal axis). There are nine GO-based clusters named *G*1,...,*G*9 that contain more than one gene. The GO clusters are separated by a horizontal bar in the heat map. Genes without annotations are omitted from the heat map. Overexpressed genes are shown with white or yellow color and underexpressed genes with red color.

We computed the similarities between the 58 DEGs using the Lin measure and converted the similarity matrix into distance using *d*(*x*, *y*) = 1 - Sim(*x*, *y*). Then we used agglomerative hierarchical clustering in R to generate gene clusters based on the GO distance matrix. The heat map that combines GO clusters and expression data is shown in Figure 1. GO-based clustering for the genes is visualised with a dendrogram on the left. To visualise the relationships between samples, a second dendrogram based on expression profiles is shown on the top. Using a dendrogram cutoff value of 0.35 we obtained nine clusters that are numbered *G*1,...,*G*9. The gene names for these nine clusters are given in Table 2 in the same order as Figure 1. To gain further insight into the clusters, we extracted the most informative GO terms for each cluster. These are terms that occur in every gene of the cluster (taking parent-child relationships into account) and have the largest information contents. The most informative terms for each cluster and their *IC *values are listed in Table 3. To assess the significance of the *IC *values, we computed p-values using a permutation test [16]. To obtain the p-value for a cluster with the size *k*, we generated 10000 random clusters with size *k *and computed *IC *of the most informative term in each cluster. The p-value is then the fraction of clusters having *IC *at least as great as the cluster under study.

**Table 2:** Genes corresponding to the most statistically significant clusters found in the case study.

Cluster	Genes
G1	PDCL3 MAGED1 (two probe sets) PRKCE PRDX1 CLIC4 MRPS23
	GABARAPL3
G2	CBR3 RANBP17 NBEA FVT1
G3	LRRC47 WARS
G4	NANOGP8 ZNF215 POU5F1 MYBL2 L1TD1 CITED2 TCEA2 SMARCAD1
	MKI67IP CPSF4 PPFIBP2 WDSUB1 PPP3CA ISG20L1 TIPARP CEP290
	DPPA4 TJP2 NLRP7
G5	CTH GLO1
G6	PLAU PPAP2A
G7	TLR5 OR5R1 TMEM106C IFITM1 PCDHB5 PCDHB11 AC069513.28 PLK3
G8	SLC22A17 FLVCR1
G9	PDGFA IGSF21 GDF3 CCDC80 GAL TF

*MAGED1 *has two distinct differentially expressed probe sets. Genes are ordered from bottom to top in Figure 1. For example, the genes in cluster *G*1 in Figure 1 are, from bottom to top, *PDCL3*, two probe sets for *MAGED1*, *PRKCE*, etc.

**Table 3:** Most informative GO terms for the clusters obtained from microarray data.

Cluster	Size	p-value	*IC*	GO term
G1	8	0.0063	1.835	cytoplasm
G2	4	0.20	1.835	cytoplasm
			0.888	binding
G3	2	0.009	8.751	aminoacyl-tRNA ligase activity
			5.841	translation
			0.888	binding
G4	19	0.020	0.888	binding
G5	2	0.0007	12.012	carbon-sulfur lyase activity
			1.835	cytoplasm
			1.753	primary metabolic process
G6	2	0.21	4.705	negative regulation of biological process
			3.420	hydrolase activity
			3.411	cellular protein metabolic process
G7	8	0.020	1.366	membrane
G8	2	0.25	4.238	transporter activity
			3.181	transport
			2.866	integral to membrane
G9	6	< 0.0001	3.967	extracellular region

*IC *is the information content of given term. P-values are derived using a permutation test with 10000 repetitions. At most three GO terms are shown for each cluster. Only common GO terms with *IC *> 0 are shown.

The cluster *G*5 consists of two genes: cystathionase (*CTH*) and glyoxalase I (*GLO1*). These two genes correlate strongly in their GO terms as their extremely high IC-value of 12.0 indicates. Also their gene expression patterns are almost identical across the samples as shown in the heat map in Figure 1. *GLO1 *is a glutathione-binding protein that contributes to several pathways that are associated with various diseases, such as cancers [22]. As glutathione plays a key role in the process where tumor cells acquire resistance to anti-cancer drugs, *GLO1 *inhibitors are considered as potential anti-cancer agents [22,23]. *CTH *is a critical factor in glutathione synthesis and has recently been associated with increased risk of bladder cancer [24]. While detailed discussion of the exact roles of *CTH *and *GLO1 *in embryonal carcinomas is out of scope of this study, our results suggest that *GLO1 *and *CTH *may function in concert, and contribute to tumor progression and drug resistance in embryonic cancers.

Interestingly, *CTH *and *GLO1 *contribute to the same biological process but do not have common pathways in the KEGG pathway database [25] as shown in Table 4 that contains all KEGG pathways associated to genes in Table 2. Thus, *CTH *and *GLO1 *would not have been grouped together with standard pathway analyses despite the fact that, based on literature, their biological function is markedly similar.

**Table 4:** KEGG pathways for differentially expressed genes.

Cluster	Gene	KEGG pathways
G1	GABARAPL3	Regulation of autophagy
G1	PRKCE	Tight junction, Fc epsilon RI signaling pathway, Type II diabetes mellitus
G2	FVT1	Sphingolipid metabolism
G2	CBR3	Arachidonic acid metabolism
G3	WARS	Tryptophan metabolism, Aminoacyl-tRNA biosynthesis
G4	PPP3CA	MAPK signaling pathway, Calcium signaling pathway, Apoptosis, Wnt signaling pathway, Axon guidance, VEGF signaling pathway, Natural killer cell mediated cytotoxicity, T cell receptor signaling pathway, B cell receptor signaling pathway, Long-term potentiation, Amyotrophic lateral sclerosis (ALS)
G4	TJP2	Tight junction, Vibrio cholerae infection
G5	CTH	Glycine, serine and threonine metabolism, Methionine metabolism, Cysteine metabolism, Selenoamino acid metabolism, Nitrogen metabolism
G5	GLO1	Pyruvate metabolism
G6	PLAU	Complement and coagulation cascades
G6	PPAP2A	Glycerolipid metabolism, Glycerophospholipid metabolism, Ether lipid metabolism, Sphingolipid metabolism
G7	TLR5	Toll-like receptor signaling pathway, Pathogenic Escherichia coli infection -EHEC and EPEC
G7	OR5R1	Olfactory transduction
G7	IFITM1	B cell receptor signaling pathway
G9	PDGFA	MAPK signaling pathway, Focal adhesion, Gap junction, Regulation of actin cytoskeleton, Glioma, Prostate cancer, Melanoma

Genes not shown in the table did not have any KEGG pathway annotation.

## Conclusion

We have developed tools to cluster genes from microarray experiments using semantic similarity measures. Using benchmark tests we demonstrated clear speed gain as compared to existing implementations. Our efficient implementation of similarity measures enables analysis of gene sets with hundreds of genes that are typically seen in microarray experiments. We then combined expression data and GO annotations using hierarchical clustering and a heat map visualisation that together enable rapid identification of genes sharing similar biological functions. In our case study we further analysed genes that are differentially expressed in testicular germ cell tumors between undifferentiated embryonal carcinomas and differentiated testicular cells. Our results suggest that GO-based annotation analysis approaches may be able to take advantage of the accumulated knowledge available in literature over approaches using pathway databases, which are typically updated in a much slower pace than the GO database. In summary, the csbl.go package allows rapid visualisation of gene GO and expression profiles, and thereby facilitates hypothetisising gene functions in cells.

## Availability and requirements

• **Project name**: csbl.go

• **Project home page**: 

• **Operating system(s)**: Platform independent; tested on Windows and Linux

• **Programming language**: R (version 2.6 or greater)

• **License**: GNU General Public License

## Competing interests

The authors declare that they have no competing interests.

## Authors' contributions

KO implemented the semantic similarity measures and GO based clustering functionality, and wrote the manuscript. ML implemented the heat map visualisation, provided a tool to retrieve Ensembl annotations and critically commented the manuscript. SH coordinated the study and was involved in writing the manuscript. All authors read and approved the final manuscript.

## References

[B1] Ashburner M, Ball C, Blake J, Botstein D, Butler H, Cherry J, Davis A, Dolinski K, Dwight S, Eppig J, Harris M, Hill D, Issel-Tarver L, Kasarskis A, Lewis S, Matese J, Richardson J, Ringwald M, Rubin G, Sherlock G (2000). Gene ontology: tool for the unification of biology. The Gene Ontology Consortium. Nat Genet.

[B2] Guo X, Liu R, Shriver C, Hu H, Liebman M (2006). Assessing semantic similarity measures for the characterization of human regulatory pathways. Bioinformatics.

[B3] Pesquita C, Faria D, Bastos H, Ferreira A, Falcão A, Couto F (2008). Metrics for GO based protein semantic similarity: a systematic evaluation. BMC Bioinformatics.

[B4] Brun C, Chevenet F, Martin D, Wojcik J, Guenoche A, Jacq B (2004). Functional classification of proteins for the prediction of cellular function from a protein-protein interaction network. GENOME BIOLOGY.

[B5] Huang D, Sherman B, Tan Q, Collins J, Alvord W, Roayaei J, Stephens R, Baseler M, Lane H, Lempicki R (2007). The DAVID Gene Functional Classification Tool: a novel biological module-centric algorithm to functionally analyze large gene lists. Genome Biol.

[B6] Couto FM, Silva MJ, Coutinho PM (2007). Measuring semantic similarity between Gene Ontology terms. Data Knowl Eng.

[B7] Lord P, Stevens R, Brass A, Goble C (2003). Investigating semantic similarity measures across the Gene Ontology: the relationship between sequence and annotation. Bioinformatics.

[B8] Resnik P (1995). Using information content to evaluate semantic similarity in a taxonomy. Proceedings of the 14th International Joint Conference on Artificial Intelligence.

[B9] Lin D (1998). An information-theoretic defiition of similarity. Proceedings of the 15th International Conference on Machine Learning.

[B10] Jiang J, Conrath D (1997). Semantic similarity based on corpus statistics and lexical taxonomy. Proceedings of International Conference on Research in Computational Linguistics.

[B11] Schlicker A, Domingues F, Rahnenführer J, Lengauer T (2006). A new measure for functional similarity of gene products based on Gene Ontology. BMC Bioinformatics.

[B12] Frohlich H, Speer N, Poustka A, Beißbarth T (2007). GOSim-An R-package for computation of information theoretic GO similarities between terms and gene products. BMC Bioinformatics.

[B13] Bodenreider O, Aubry M, Burgun A (2005). Non-lexical approaches to identifying associative relations in the Gene Ontology. Pacific Symposium on Biocomputing Pacific Symposium on Biocomputing.

[B14] R Development Core Team (2007). R: A Language and Environment for Statistical Computing.

[B15] Anduril framework. http://csbi.ltdk.helsinki.fi/anduril/.

[B16] Good P (2000). Permutation tests: a practical guide to resampling methods for testing hypotheses.

[B17] Skotheim R, Lind G, Monni O, Nesland J, Abeler V, Fossa S, Duale N, Brunborg G, Kallioniemi O, Andrews P, Lothe R (2005). Differentiation of human embryonal carcinomas in vitro and in vivo reveals expression profiles relevant to normal development. Cancer Research.

[B18] SemSim package. http://bioconductor.org/packages/2.1/bioc/html/SemSim.html.

[B19] Draghici S (2003). Data Analysis Tools for DNA Microarrays.

[B20] Pounds S, Cheng C (2006). Robust estimation of the false discovery rate. Bioinformatics.

[B21] Hubbard T, Barker D, Birney E, Cameron G, Chen Y, Clark L, Cox T, Cuff J, Curwen V, Down T, Durbin R, Eyras E, Gilbert J, Hammond M, Huminiecki L, Kasprzyk A, Lehvaslaiho H, Lijnzaad P, Melsopp C, Mongin E, Pettett R, Pocock M, Potter S, Rust A, Schmidt E, Searle S, Slater G, Smith J, Spooner W, Stabenau A (2002). The Ensembl genome database project. Nucleic Acids Research.

[B22] Laga M, Cottyn A, Van Herreweghe F, Berghe W, Haegeman G, Van Oostveldt P, Vandekerckhove J, Vancompernolle K (2007). Methylglyoxal suppresses TNF-α-induced NF-κB activation by inhibiting NF-κB DNA-binding. Biochemical Pharmacology.

[B23] Balendiran G, Dabur R, Fraser D (2004). The role of glutathione in cancer. Cell Biochemistry And Function.

[B24] Moore L, Malats N, Rothman N, Real F, Kogevinas M, Karami S, Garcia-Closas R, Silverman D, Chanock S, Welch R, Tardffon A, Serra C, Carrato A, Dosemeci M, García-Closas M (2007). Polymorphisms in one-carbon metabolism and trans-sulfuration pathway genes and susceptibility to bladder cancer. Int J Cancer.

[B25] Kanehisa M, Goto S (2000). KEGG: Kyoto Encyclopedia of Genes and Genomes. Nucleic Acids Research.

